# Dendritic-Tumor Fusion Cells Derived Heat Shock Protein70-Peptide Complex Has Enhanced Immunogenicity

**DOI:** 10.1371/journal.pone.0126075

**Published:** 2015-05-11

**Authors:** Yunfei Zhang, Yong Zhang, Jun Chen, Yunyan Liu, Wen Luo

**Affiliations:** 1 Department of Orthopaedic, Orthopaedic Oncology Institute of PLA, Tangdu Hospital, Fourth Military Medical University, Xinsi Road, Xi’an, Shaanxi Province, China; 2 Department of Ultrasound, Xijing Hospital, Fourth Military Medical University, Changle Road, Xi’an, Shaanxi Province, China; Cedars-Sinai Medical Center, UNITED STATES

## Abstract

Tumor-derived heat shock protein70-peptide complexes (HSP70.PC-Tu) have shown great promise in tumor immunotherapy due to numerous advantages. However, large-scale phase III clinical trials showed that the limited immunogenicity remained to be enhanced. In previous research, we demonstrated that heat shock protein 70-peptide complexes (HSP70.PC-Fc) derived from dendritic cell (DC)-tumor fusions exhibit enhanced immunogenicity compared with HSP70.PCs from tumor cells. However, the DCs used in our previous research were obtained from healthy donors and not from the patient population. In order to promote the clinical application of these complexes, HSP70.PC-Fc was prepared from patient-derived DC fused directly with patient-derived tumor cells in the current study. Our results showed that compared with HSP70.PC-Tu, HSP70.PC-Fc elicited much more powerful immune responses against the tumor from which the HSP70 was derived, including enhanced T cell activation, and CTL responses that were shown to be antigen specific and HLA restricted. Our results further indicated that the enhanced immunogenicity is related to the activation of CD4+ T cells and increased association with other heat shock proteins, such as HSP90. Therefore, the current study confirms the enhanced immunogenicity of HSP70.PC derived from DC-tumor fusions and may provide direct evidence promoting their future clinical use.

## Introduction

Numerous preclinical and clinical studies have shown that tumor-derived heat shock protein-peptide complexes (HSP.PC) can induce antitumor immune responses [1,2,3,4]. Vaccination with tumor derived GP96, HSP70 or HSP90 can induce protective immunity against the tumors challenge used as the source of the HSPs in animal studies[5,6]. In addition, effective treatment including reducing of tumor burden and inhibition of metastasis can also be induced [7,8]. The results from clinical trials (including phase III) proved effective tumor-specific immune responses can be induced by HSP.PC derived from tumor [2, 9,10,11,12,13,14]. These preclinical and clinical results demonstrate the potential of tumor derived HSP.PC in tumor immunotherapy.

However, the effectiveness of tumor derived HSP.PC require further improvement. As immunotherapy strategy against established tumors, it is only marginally effective, especially when widely metastatic diseases were treated[15]. In rodent models, when minimal diseases were treated, it was highly effective [7,16]; however, when widely metastatic diseases were treated, only a small proportion of treated animals showed benefits[7,17,18,19]. Large-scale phase III clinical trials demonstrated two major disadvantages of tumor-derived HSP70.PC[12, 13]; first, the immunogenicity was inadequate and even negative results was observed as Christopher Wood et al has described[13]; second, the vaccine source was sometimes limited. Thus, the development of novel tumor-derived HSP vaccines with improved immunogenicity is required.

Previous studies have shown that HSP70.PC derived from DC-tumor fusions (HSP70.PC-Fc) have enhanced immunogenicity compared with those derived from tumor cells (HSP70.PC-Tu). The enhanced immunogenicity of HSP70.PC-Fc was associated with optimization of composition including increased content of antigenic tumor peptides and other heat shock proteins, such as HSP90. These results suggest an alternative approach to prepare HSP-based vaccines using DC-tumor fusion technology. In the previous study, however, HSP70.PC-Fc was obtained from healthy donor-derived DC fused with tumor cells. To promote the clinical application, in this study, HSP70.PC was prepared from fusions of patient-derived DC and tumor cells. Our results show that HSP70.PC derived from DC-tumor fusion cells can elicit much more powerful antitumor immune responses against autologous tumor cells than those from tumor cells. Consistent with previous reports[20], the enhanced immunogenicity is related to activation of CD4+ T cells and increased association with other heat shock proteins such as HSP90. These results provide additional evidence in support of the potential use of HSP70.PC derived from DC-tumor fusion cells in a clinical setting.

## Materials and Methods

### Ethics statements

The experimental protocol involved the use of human peripheral blood and tumor tissue. The study protocol was approved by the IRB for human participants of the Fourth Military Medical University and Tangdu Hospital. All the participants provided their written informed consent to participate in this study and Ethics Committees of Fourth Military Medical University approved this consent procedure.

#### 1. Generation of monocyte-derived DCs and T cells

Peripheral blood mononuclear cells (PBMCs) were isolated from peripheral blood samples (80–120ml) obtained from patients with breast tumors. PBMC were isolated by Ficoll-Paque (GE Healthcare Biosciences AB, Uppsala, Sweden) density gradient centrifugation and plated in tissue culture flasks (BD Biosciences) in RPMI 1640 culture media (Sigma-Aldrich) containing 2 mM L-glutamine (Sigma-Aldrich) and supplemented with heat-inactivated 10% human AB serum (Sigma-Aldrich, 100 U/ml penicillin and 100 μg/ml streptomycin (Sigma-Aldrich) for 2 h at 37°C in a humidified 5% CO2 incubator. The monocyte-enriched adherent fraction was cultured in complete medium containing GM-CSF (1,000 U/ml) (R&D Systems, Minneapolis,MN) and IL-4 (1,000 U/ml) (R&D Systems, Minneapolis,MN) for 5 days to generate immature DCs. Non-adherent cells were frozen with 10% DMSO in human AB serum and used as a source of T cells.

#### 2. Preparation of tumor cell lines

The human breast carcinoma cell lines MCF-7 (HLA-A2+/A11- HER2+/MUC1+), SKBR3 (HLA-A2-/A11+ HER2+/MUC1+), BT20 (HLA-A2-/A11- HER2+/MUC1+) and the human myelogenous leukemia cell line K562 (HLA-A2-/A11- HER2-/MUC1-) were obtained from the American Type Culture Collection (ATCC). MCF7 cells and BT20 were grown in DMEM and EMEM, respectively; SKBR3 and K562 cells were grown in RPMI medium 1640. All media was supplemented with 10% heat-inactivated FCS (Sigma-Aldrich), 2 mM L-glutamine, and 100 U/ml penicillin and 100 μg/ml streptomycin.

#### 3. Isolation of patient-derived breast carcinoma cells

Primary breast carcinoma cells were obtained from fresh tumor tissues obtained from patients during surgery without perturbation of regular treatment. Single cell suspensions were obtained by processing solid tumor samples under sterile conditions at room temperature using a previously described method[20]. Briefly, the surgical specimens were mechanically minced into small pieces no larger than 1–3 mm^3^ in RPMI 1640 and washed twice with RPMI 1640. The small pieces of minced tumor were then placed into 250-ml trypsinizing flasks containing 30 ml of enzyme solution (1 mg/ml collagenase Type I (Sigma-Aldrich) and 0.1 mg/ml DNase (Sigma-Aldrich) in Ca++/Mg++ free Hank’s balanced salt solution pH 7.3–7.5). Enzymatically dissociated tumor tissue was then filtered through 150-μm nylon mesh to generate a single cell suspension. Tumor cells were cultured in RPMI 1640 medium supplemented with 10% heat-inactivated human AB serum. The cells were used as fusion partners, as targets in CTL assays, and for extraction of the tumor-derived HSP70-peptide complex (HSP70.PC).

#### 4. Preparation of DC and breast tumor cell fusions

DC-tumor fusion cells were prepared as previously described[20]. Tumor cells were mixed with DC preparations at 1:10 and washed in serum-free pre-warmed RPMI 1640 culture medium. The cell pellet was resuspended in 50% PEG solution (molecular mass: 1,450) (Sigma-Aldrich). After 3 min at room temperature, the PEG solution was progressively diluted over the subsequent 5 min with pre-warmed serum-free RPMI 1640 medium. After washing with serum-free RPMI 1640, fused cells were resuspended in RPMI 1640 medium supplemented with 10% human AB serum and 500 U/ml GM-CSF and cultured in 5% CO2 at 37°C for 5 days by which time each DC-tumor fusion cells had become integrated into a single entity that was loosely adherent to the culture dish.

#### 5. Characterization of patient-derived breast tumor cells, patient-derived DCs and DC-tumor fusion cells

Patient-derived breast carcinoma cells and patient-derived DCs were incubated with primary mouse anti-human mAbs against the tumor antigens MUC1, HER2, HLA-A2, HLA-A11, HLA-DR and CD86 and matched isotype controls (BD Pharmingen). Cells were then washed, and incubated with FITC-conjugated mouse anti-mouse IgG1 (BD Pharmingen). DC-tumor fusion cell preparations were subjected to dual staining to quantify the percentage of cells that co-expressed the unique DC marker (HLA-DR-PE) and the tumor marker (HER2-FITC) (BD Pharmingen). Cells were fixed in 2% paraformaldehyde (Sigma-Aldrich) and underwent flow cytometry analysis using FACScan (BD Biosciences) and CellQuest software (BD Biosciences). True fusion cells were identified by immunofluorescence staining and laser confocal microscopy. Approximately 2×10^4^ DC-tumor fusion cells were spun onto slides (Cytospin 4 Thermo Shandon), allowed to dry, and fixed with 4% paraformaldehyde for 8 min. The slides were incubated with FITC-conjugated mouse anti-human HER2 mAb for 1 h at 4°C. The slides were then washed and incubated with PE-conjugated mouse anti-human HLA-DR mAb for 1 h at 4°C before being washed, fixed in 4% paraformaldehyde, and analyzed by confocal microscopy (Nikon Eclipse TE2000-E).

#### 6. Immunoprecipitation of HSP70-peptide complexes from DC-tumor fusion cells and tumor cells

The HSP70.PC was purified by immunoprecipitation with rabbit anti-human HSP70 mAb (Abcam) as described previously[20]. Briefly, DC-tumor fusion cells and tumor cells were collected and washed three times with ice cold phosphate-buffered saline (PBS, pH 7.4). Then cells were incubated in lysis buffer containing a protease inhibitor cocktail (Roche) (50 mM Tris-HCl, pH 7.4, 50 mM NaCl, 1% (v/v) Nonidet P-40, 1 mM NaV) on ice for 30 min, then centrifuged at 13,000 rpm for 15 min. The mAb against HSP70 (Abcam, Burlingame, California) was added to the supernatant at a concentration of 10 μg/ml and the mixture was gently rotated at 4°C overnight. Then, 80 μl protein A/G-sepharose (1:1) (Sigma-Aldrich) was added and incubated at 4°C for an additional 90 min. The protein sepharose was collected by centrifugation and after extensive washing in lysis buffer, the immunoprecipitates were eluted into high salt PBS (500 mM NaCl). The concentration of protein in the eluate was determined for use in lymphocyte stimulation.

#### 7. Immunoblotting

For determination of the immunoprecipitated proteins, the bound proteins were released from the protein A/G-sepharose beads by adding 2× SDS gel loading buffer and boiling for 5 min. Following centrifugation, equal volumes of each sample were fractionated by SDS-PAGE. Following electrophoresis, proteins were transferred to nitrocellulose membranes, which were incubated with primary antibodies at the indicated concentrations for 2 h at 27°C. Subsequently, membranes were washed, and incubated with horseradish peroxidase-labeled secondary antibody (1:4,000; Amersham Biosciences) for 1 h at 27°C. Protein bands were visualized using an enhanced chemiluminescence (ECL) Detection System (GE) according to the manufacturer’s instructions. A panel of antibodies against HSP110, HSP90, HSP70 (Abcam Burlingame, California) were used. Quantification of protein bands was achieved by densitometric analysis using ChemilmagerTM 5500 (Alpha Innotech). The optical density (OD) of a protein band in a sample was expressed as relative optical density (ODr) according to the amount of protein loaded into the corresponding well. Each blot was carried out in triplicate. To avoid errors due to differences in signal intensity between the three blots, the ODr were normalized as follows: for each protein, the ODr of all samples on each blot was summed (Si = ∑ ODr, on blot i). The ODr of each band on the blot was then normalized according to the mean of these sums: normODr = (S1 + S2 + S3)/3Si.

#### 8. T cell activation by HSP70.PC derived from DC-tumor fusion cells

T cell activation by HSP70.PC-Fc was investigated by IFN-γ enzyme-linked immunosorbent spot assays (IFNγ ELISPOT kit Diaclone, Besancone, France) and intracellular IFN-γ staining of CD4 and CD8 T cells. Briefly, non-adherent PBMCs purified by the nylon wool method were co-cultured with HSP70.PC-Tu or HSP70.PC-Fc in medium containing 10% human AB serum in the presence of 10 U/ml human IL-2 for 5 days; medium alone was used as control. The stimulated T cells were then harvested by separation on a nylon wool column for use as effector cells and autologous breast tumor cells were used as stimulator cells. Stimulator cells (5×10^5^) and effector cells (5×10^5^) were plated on PVDF-bottomed 96-well plates coated with anti-IFN-γ antibody. After incubation at 37°C for 24 h, the cells were removed and a biotinylated IFN-γ detection antibody was added for 2 h. Free antibody was washed off, and the plates were incubated with streptavidin-alkaline phosphatase for 1 h at 37°C. Streptavidin-alkaline phosphatase bound to biotin was detected via BCIP/NBT (Bio-Rad) substrate. Resulting spots were counted with a stereomicroscope under magnification of 20× to 40×. Medium control, as well as tumor cells without effector cells, was included as negative control. Intracellular IFN-γ staining was performed to further analyze the IFN-γ expression of CD4 and CD8 T cells. T cells were first stained with mAbs against CD4 or CD8 (BD Pharmingen), followed by membrane permeabilization, then stained with mAbs against IFN-γ (BD Pharmingen). Cells were washed and fixed with 2% paraformaldehyde, and subjected to flow cytometric analysis by FACScan using CellQuest software.

#### 9. CTL responses by HSP70.PC derived from DC-tumor fusion cells

Analysis of autologous breast tumor cell cytotoxicity induced by HSP 70.PC-Fc was performed with CytoTox 96 Non-Radioactive Cytotoxicity Assay kits (Promega Inc., WI, USA) according to the manufacturer’s instructions. Briefly, lymphocytes were stimulated with HSP 70.PC-Tu or HSP 70.PC-Fc as described in T cells activation section. After 5 days of incubation, the stimulated T cells were harvested by separation on a nylon wool column and used as effector cells in CTL assays. Autologous breast tumor target cells were co-cultured with effector T cells for 4 h at effector:target cell ratios of 12.5:1, 25:1, and 50:1. After centrifugation at 500 ×g for 5 min, 50 μl aliquots of supernatant were transferred to fresh 96-well flat-bottom plates, and an equal volume of reconstituted substrate mix was added to each well. The plates were incubated at room temperature for 30 min and protected from light. Then 50 μl stop solution was added, and the absorbance values were measured at 492 nm. The percentage of cytotoxicity for each effector:target cell ratio was calculated according to the following equation: [A (experimental) − A (effector spontaneous) − A (target spontaneous)]×100/ [A (target maximum) − A (target spontaneous)]. To assess the function of activated DCs in the CTL responses, the DCs mixed with tumor were included as control in patient4 and patient5 and the effector:target ratio was 50:1.

To assess the tumor antigen specificity and MHC restriction of CTLs induced by HSP 70.PC-Fc, autologous monocytes and allogeneic tumor cell lines, including human breast carcinoma cell line MCF-7 (HLA-A2+/A11- HER2+/MUC1+), SKBR3(HLA-A2-/A11+ HER2+/MUC1+), BT20 (HLA-A2-/A11- HER2+/MUC1+) and K562 (HLA-A2-/A11- HER2-/MUC1-) were also used as targets in a parallel CTL assay as controls with an effector:target cell ratio of 50:1.

#### 10. Statistical analysis

All data were presented as the means ± SD. One-way ANOVA and LSD t-tests were used to determine the difference within each group for Western blot assays, CTL, IFN-γ ELISPOT and IFN-γ expression assays. P < 0.05 was considered significant.

## Results

### 1. Characterization of patient-derived breast tumor cells, DC and DC-tumor fusion cells

**Fig 1:**
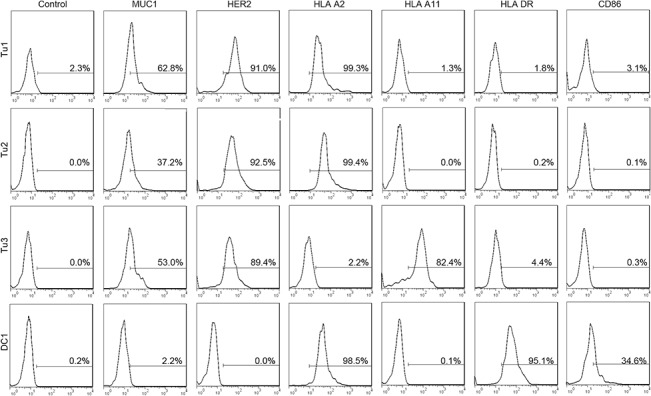
Characterization of patient-derived DCs and autologous breast tumor cells. Phenotype of patient-derived auto breast tumor cells (from Patient 1, Patient 2, Patient 3). Tumor cells were stained with antibodies against tumor antigens MUC1, HER2, HLA-A2, HLA-A11 and HLA-DR, CD86 and analyzed by flow cytometry. Phenotype of patient-derived DCs (from Patient 1). DCs were stained with antibodies against MUC1, HER2, HLA-A2, HLA-A11, HLA-DR, and CD86.

**Fig 2:**
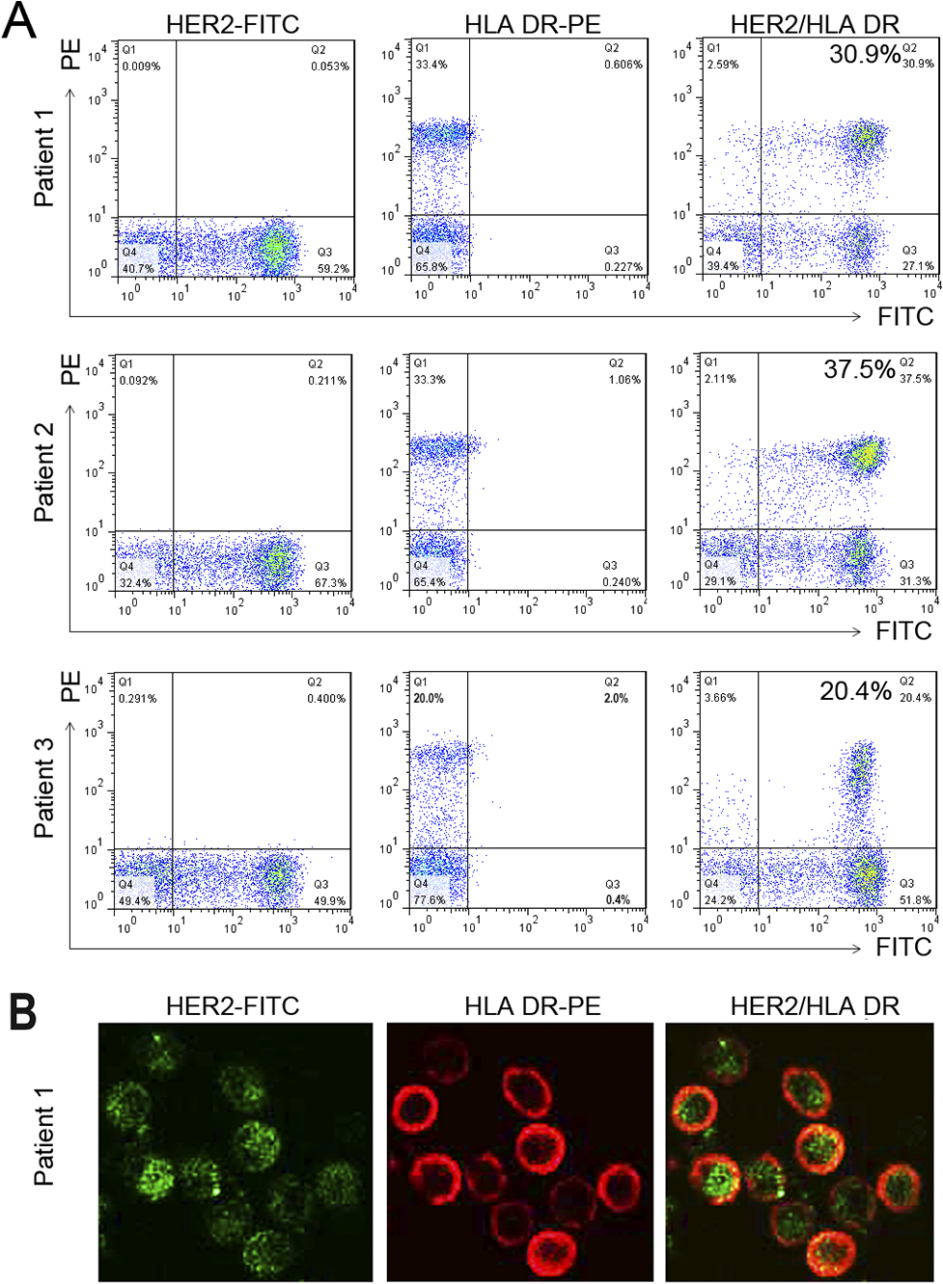
Characterization of DC-tumor fusion cells. A, Fusion cells were double-stained for the unique DC marker (HLA-DR-PE) and the tumor marker (HER2-FITC) and analyzed by 2-color flow cytometry to quantify the percentage of double-positive fusion cells. B, Identification of DC-tumor fusion cells by laser confocal microscopy. DC-tumor fusion cells (cytospin on the slides) were identified directly by observation of HER2-FITC and HLA-DR-PE under confocal microscopy. Left panel, HER2-FITC positive; middle panel, HLA-DR-PE; right panel, merged image showing double-positive fusion cells.

Patient-derived breast tumor cells expressed high levels of the tumor antigens MUC1 and HER2/neu and HLA-A*0201+ or HLA-A*0211+ (Fig 1). Patient DCs expressed high levels of HLA-DR and CD86 (Fig 1). After fusion with PEG and 3 days of culture, fusion efficiency was determined by dual color flow cytometric analysis of fusion cell expression of the DC marker HLA-DR and the tumor marker HER2/neu. The fusion efficiency was 30.9%, 37.5% and 20.4% for samples from Patients 1, 2 and 3, respectively (Fig 2A). Successful fusion was confirmed by the observation of individual cells that were positive for both HER2/neu and HLA-DR by confocal microscopy (Fig 2B).

### 2. Enhanced association of HSP90 in HSP70.PC derived from DC-tumor fusion cells

**Fig 3:**
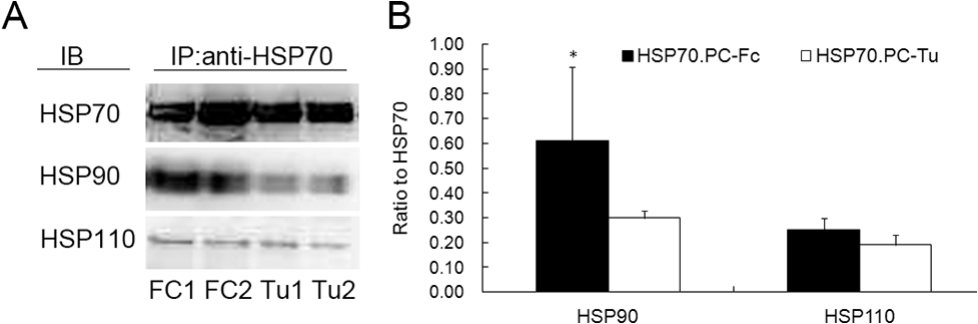
Characterization of HSP70.PC derived from tumor cells and DC-tumor fusion cells. A, Association of HSP proteins in HSP70.PC-Tu and HSP70.PC-Fc. Lysates from patient-derived breast tumor cells and from DC-tumor fusion cells were immunoprecipitated with anti-HSP70 mAb followed by immunoblotting with mAbs against HSP110 and HSP90; HSP70 was used as a loading control (results from Patient 1 and Patient 2 are shown). B, The results are representative of three independent blots. The relative protein density was determined by densitometric analysis and normalized between different blots (The ODr (relative optical density) of each band on the blot was normalized according to the mean of these sums: normODr = (S1 + S2 + S3)/3Si.). The statistical significance for the ratio of HSP110, HSP90, to HSP70 between HSP70.PC-Tu and HSP70.PC-Fc was determined by Student’s t-test (* indicates P<0.05).

In order to determine whether other HSPs were associated with HSP70.PC, HSP70 was immunoprecipitated with anti-HSP70 mAb followed by blotting with HSP110, HSP90 or HSP70. HSP110, and HSP90 were detected in HSP70.PC derived from both tumor and DC-tumor fusion cells (Fig 3A). However, the content of HSP90 was significantly increased in HSP70.PC-Fc compared with that derived from tumor cells. The difference in HSP90 content in HSP70.PC derived from tumor and fusion cells was statistically significant. P<0.05 (Fig 3B).

### 3. Enhanced T cell activation by HSP70.PC derived from DC-tumor fusion cells

**Fig 4:**
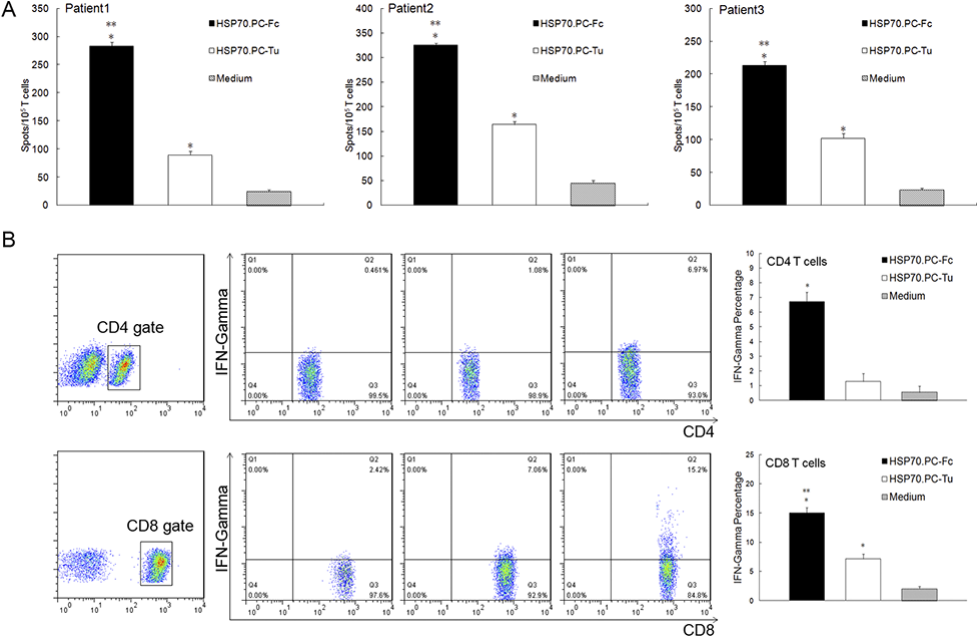
Activation of T cells by HSP70.PC derived from DC-tumor fusion cells. Indicated numbers of lymphocytes and DCs (10:1 ratio) were incubated with HSP70.PC-Tu (2 μg/ml) or HSP70.PC-Fc (2 μg/ml) in medium containing 10% human AB serum in the presence of 10 U/ml human IL-2. Medium alone was used as a control. After 5 days of incubation, the stimulated T cells were harvested by nylon wool separation. A, ELISPOT enumeration of IFN-γ producing cells. B, Intracellular expression of IFN-γ in CD4 and CD8 cells (data from Patient 1 shown). The statistical significance was determined by one-way ANOVA (* indicates P<0.05, HSP70.PC vs. medium, ** indicates P<0.05, HSP70.PC-Tu vs. HSP70.PC-Fc).

To determine T cell activation induced by HSP70.PC-Fc, IFN-γ production was measured by ELISPOT assays and intracellular staining. As shown in Fig 4A, significantly greater numbers of T cells stimulated with both HSP70.PC-Fc and HSP70.PC-Tu produced IFN-γ compared with T cells cultured with medium alone. The IFN-γ secretion was much higher in T cells stimulated with HSP70.PC-Fc than those stimulated with HSP70.PC-Tu (P<0.05). Similar results were observed by intracellular staining. Although both HSP70.PC-Fc and HSP70.PC-Tu induced significant expression of IFN-γ in T cells compared with those cultured with medium alone (Fig 4B), the highest level of IFN-γ expression was observed in both CD4 and CD8 T cells stimulated by Hsp70.PC from fusion cells (Fig 4B, P<0.05).

### 4. Enhanced CTL activity stimulated by HSP70.PC derived from DC-tumor fusion cells

**Fig 5:**
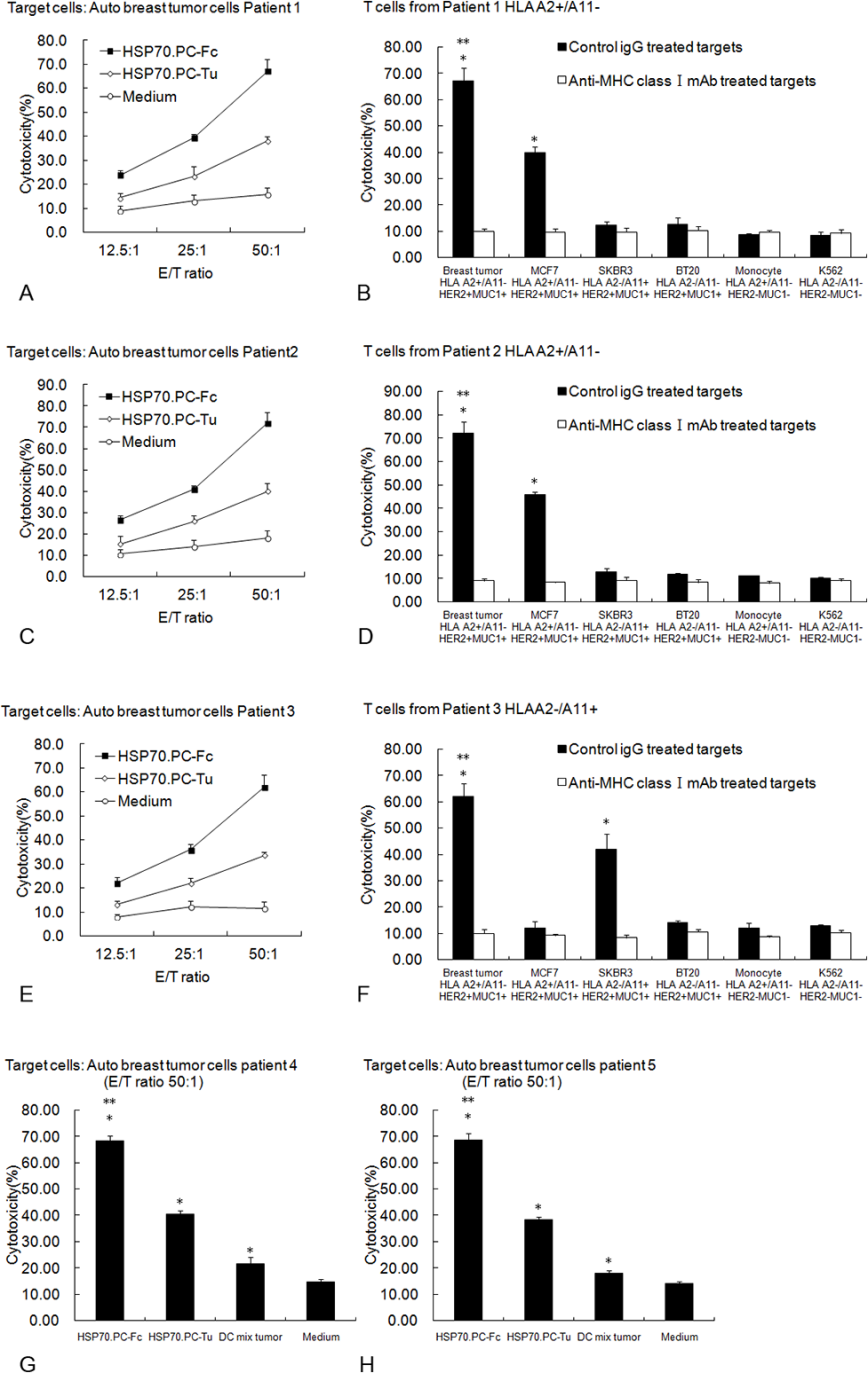
CTL responses against tumor stimulated by HSP70.PC derived from DC-tumor fusion cells. The indicated number of lymphocytes and DCs (10:1 ratio) were incubated with HSP70.PC-Tu or HSP70.PC-Fc (2 μg/ml) in medium containing 10% human AB serum in the presence of 10 U/ml human IL-2. Medium alone was used as a control. After 5 days of incubation, the stimulated T cells were harvested by nylon wool separation for use as effector cells. CTL activity against autologous breast tumor cells was analyzed by LDH release assay (A from Patient 1 [HLA-A2+/A11-], C from Patient 2 [HLA-A2+/A11-], E from Patient 3 [HLA-A2-/A11+]). DC mixed with tumor were included as control in Patient 4 (G) and Patient 5 (H). (*indicates P<0.05, HSP70.PC-Fc, HSP70.PC-Tu, DC mix tumor vs. Medium. ** indicates P<0.05, HSP70.PC-Fc vs. HSP70.PC-Tu, DC mix tumor or Medium). In order to test the tumor specificity of CTL responses, different tumor targets with different HLA phenotypes (breast tumor cell line MCF7 HLA-A2+ A11-, SKBR3 HLA-A2- A11+, BT20 HLA-A2- A11-, leukemia cell line K562 HLA-A2- A11-, autologous monocytes) were included in CTL assays (B from Patient 1, D from Patient 2, F from Patient 3). All determinations were conducted in triplicate and expressed as the mean ± SD. The statistical significance was determined by one-way ANOVA. (*indicates P<0.05, target vs. target treated with anti-MHC class antibody. ** indicates P<0.05, autologous breast tumor cells vs. MCF7 or SKBR3).

CTL activity against breast tumor cells was determined in LDH release assays. Enhanced CTL activity was observed in T cells stimulated with HSP70.PC-Fc. As shown in Fig 5A, 5C and 5E, T cells stimulated with both HSP70.PC-Tu and HSP70.PC-Fc mediated significantly more autologous breast tumor cell lysis compared with those cultured with medium alone; the highest level of lysis was found to be mediated by T cells stimulated with HSP70.PC-Fc (P<0.05). As Fig 5G and 5H showed the effective CTL activity against breast tumor cells can also be induced by DCs mixed with tumor control compared with medium control.

T cell specificity was investigated using a panel of breast cancer cells including autologous breast cancer cells, autologous monocytes and allogeneic tumor cell lines as targets. The highest level of lysis was observed against autologous breast cancer cells followed by the lysis of targets that shared expression of tumor antigens and HLA elements. As shown in Fig 5B and 5D, effector T cells (HLA A2+ from Patient 1 or Patient 2) stimulated with HSP70.PC-Fc lysed the autologous breast cancer cells (HLA A2+) and to a lesser extent, the MCF7 breast tumor cells (HLA A2+, MUC1+, HER2+). The lysis of SKBR3 (HLA A2-, MUC1+, HER2+) or BT20 (HLA A2-, MUC1+, HER2+) was minimal. Similar results were observed for Patient 3 (HLA A11+). The T cells stimulated with HSP70.PC-Fc lysed the autologous breast cancer cells (HLA A11+) with the greatest efficacy followed by lysis of SKBR3 that shared tumor antigens and HLA elements (HLA A11+/A2-, MUC1+, HER2+). In addition, lysis of the targets was significantly reduced by preincubation of the tumor cells with anti-HLA-ABC mAb (Fig 5B, 5D and 5F). The lysis of K562 and autologous monocytes was minimal. These results indicate that the CTL activity induced by HSP70.PC-Fc is tumor antigen specific and MHC Class I-restricted.

## Discussion

As previous described that[21], currently, there were more than 150 medical institutions undertaking basic and clinical research on HSPs. The two largest randomized, open-label, multicenter phase III clinical trials reported in 2008 further confirmed that HSP-based vaccines were safe and clinically feasible [12,13], which has inspired further research. However, these phase III clinical trials also showed the limitations of HSP-based vaccines. First, the efficacy of the current vaccines is usually observed in early-stage disease or with high-dose vaccination, demonstrating lack of immunogenicity. Second, approximately half of patients do not have adequate amount of tumor cells for the preparation of sufficient vaccine; thus, the clinical use of HSP vaccines was limited by the yield of tumor tissues [12,13]. Consequently, the enhancement of immunogenicity of these vaccines is urgently needed[22].

Some alternatives have been tested, including HSPs-pulsed dendritic cells[15], chaperone-rich cell lysates derived from tumors(CRCL) [23], combination with GM-CSF [18, 24], recombinant HSPs with tumor-associated peptides[25] or encapsulated in nanoemulsions [26] and have shown improved immunogenicity. Furthermore, improved antitumor immune responses have been observed following the development of specific new methods of vaccine preparation for enhanced immunogenicity[27,28].

The generation of HSP70.PC from DC-tumor fusion cells represents one of these alternatives[20]. Previous studies by Gong et al demonstrated enhanced immunogenicity of HSP70.PC-Fc compared with that of HSP70.PC-Tu in the induction of T cells proliferation and CTL responses against tumor cells[20]. However, the DCs used in previous study were obtained from healthy donors rather than from cancer patients. In order to promote the clinical application, patient-derived DCs and tumor cells were fused in this study, and the immunogenicity of HSP70.PC-Fc was analyzed.

The data presented here showed a clear advantage of the HSP70.PC vaccine extracted from DC-tumor fusion cells over comparable extracts from tumor cells alone. HSP70.PC-Fc contains HSP90 that may increase its ability to carry antigenic peptides. In addition, HSP70.PC-Fc induced enhanced CD4 and CD8 T cell responses. Most importantly, T cells from patients with breast cancer were shown to be stimulated and to lyse autologous cancer cells.

Tumor-derived HSP70.PC elicits effective CTL responses against the targets from which the HSP was purified[29, 30]. In our study, we found the DC-tumor fusion cell-derived HSP70.PC enhanced this CTL response against patient-derived tumor cells compared to the response induced by tumor-derived HSP70.PC. Furthermore, the CTL response was antigen specific (Fig 5). However, it should be noted that activated dendritic cells may also contribute cytokines or other factors that may passively augment the HSP70.PC based vaccine as Fig 5G and 5H showed. In addition to the enhanced CTL activity, Hsp70.PC-Fc also induced a significant CD4+ T cell response associated with the production of high levels of IFN-γ (Fig 4). CD4+ T cells play an important role in tumor immunotherapy[31,32].It has been improved the presentation of HSP-peptide complex by CD4+ T cells is important in tumor immunosurveillance. It has been reported that CD4+ T cells have a distinct role in tumor immunosurveillance based on cytokine production and/or cytotoxicity at tumor sites[33]. CD4+ T cells stimulate upregulation of MHC II and costimulatory molecules on APCs or the secretion of type cytokines that are critical to the priming phase. Mature DCs can be further influenced by CD4+ cells through a CD40-independent pathway that boosts their ability to activate CD8+ cells. Moreover, direct CD4-CD8 T cell communication via lymphokines further enhances CTL activation[34]. Recently, efficient tumor eradication by tumor-specific CD4+ T cells has been demonstrated in several mouse models and in melanoma patients[35–37].

The mechanisms underlying the enhanced immunogenicity of HSP70.PC-Fc remain to be identified. As we know, it is the antigenic peptide chaperoned with HSPs that determine the immunogenicity of the HSP70.PC. Integration of DCs and tumor cells through fusion inherit the antigen processing and presentation machinery from DC and possess the full set of accessory molecules necessary for antigen processing and presentation. Through this approach, multiple tumor antigenic peptides, including those yet unidentified, are processed by MHC Classes I and II pathways making fusion cells enriched in a wider repertoire of immunogenic peptides. It has been suggested that HSPs constitute a relay line in which the peptides, after generation in the cytosol by the action of proteases, are transferred from one HSP to another, until they are finally accepted by MHC class I molecules in the endoplasmic reticulum[38, 39]. So we think that there may be much more tumor peptides associated with HSPs in DC-tumor fusion cells compared with that from tumor cells (as S1 Fig showed) which can be supported by our previous study[20]. So, the combination of DC and tumor cells makes the fusion cells potentially good candidates from which to obtain the HSP peptide complexes based vaccine which may be the potential mechanisms for the enhanced immunogenicity. In addition, the association with HSP90 in HSP70.PC-Fc was greatly enhanced (2-fold) (Fig 3); thus, it can be speculated that the enhanced association of HSP90 in HSP70.PC-Fc leads to an increased antigenic peptide load that contributes to the enhanced antitumor immunity. However, whether there are other proteins which attribute to such enhancement and why DC-tumor fusion cells that allows for more HSP 90 co-isolation need further investigation.

In this study, we demonstrated for the first time the induction of T cell responses by HSP70.PC prepared from fusions of DC and tumor cells from cancer patients. The enhanced immunogenicity of HSP70.PC-Fc demonstrated in this study provides further evidence in support of their potential clinical application.

## Supporting Information

S1 Data Dataset and raw data of the CTL analysis for patient1 to patient5 and corresponding diagrams. (Including CTL against auto breast tumor cells at different effect-target ratio and CTL specificity against different tumor targets).
(XLS)

S2 Data Dataset and raw data of IFN-γ production by ELISPOT assays for patient1-patient3 and corresponding diagrams. (XLS)

S3 Data Raw data of IFN-γ production of CD4/CD8 lymphocytes by FACS. (XLS)

S4 Data Raw data for the Western blot assay. (XLS)

S1 Fig The hypothesis for the antigen presenting process in DC-tumor fusion cells. In tumor cells (left), it was suggested that HSPs constitute a relay line in which the peptides, after generation in the cytosol by the action of proteases, are transferred from one HSP to another, until they are finally accepted by MHC class I molecules in the ER (endoplasmic reticulum). DC-tumor fusion (right) integrated the Ag processing and presentation machinery from DC and rich tumor peptides from tumor. Through this approach, multiple tumor antigenic peptides, including those yet unidentified, are processed by MHC making fusion cells enriched in a wider repertoire of immunogenic peptides. So we think that there may be much more tumor peptides associated with HSPs in DC-tumor fusion cells compared with that from tumor cells.
(TIF)

S2 Fig This is the original figures for the Western blot and there are five parts: Fig. A. Original figure of Western blot for HSP70 (Lane 1–5: Fc1, Fc2, Tu1, Tu2, positive control); Fig. B. Original figure of Western blot for HSP90 (Lane 1–5: positive control, Fc1, Fc2, Tu1, Tu2); Fig. C. Original figure of Western blot for HSP110 (Lane 1–4: Fc1, Fc2, Tu1, Tu2); Fig. D. Original figure of NC membrane and marker for HSP70 Western blot; Fig. E. Original figure of NC membrane and marker for HSP90 Western blot; Fig. F. Original figure of NC membrane and marker for HSP110 Western blot.
(ZIP)

S1 Statistics Statistics methods and corresponding results for CTL responses. (PDF)

S2 Statistics Statistics methods and corresponding results for the IFN-γ production by ELISPOT assays. (PDF)

S3 Statistics Statistics methods and corresponding results of CD4/CD8 lymphocytes by FACS. (PDF)

S4 Statistics Statistics methods and corresponding results for Western blot assay. (PDF)
