# Supplementary figures and images for: Dendritic-Tumor Fusion Cells Derived Heat Shock Protein70-Peptide Complex Has Enhanced Immunogenicity
Source: PLoS One. 2015 May 11;10(5):e0126075. doi: 10.1371/journal.pone.0126075 (PMC4427282; doi:10.1371/journal.pone.0126075)

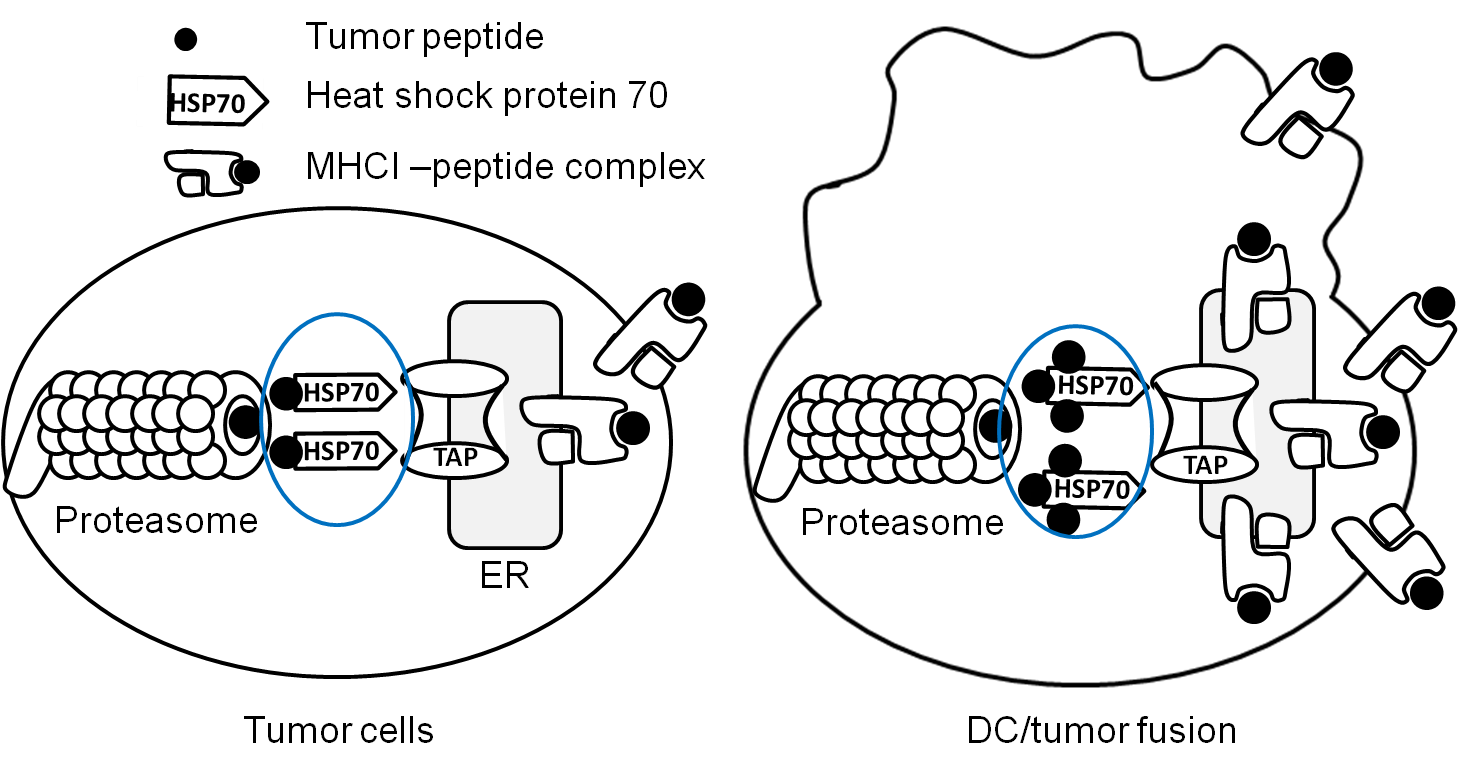

Supplement: S1 Fig — In tumor cells (left), it was suggested that HSPs constitute a relay line in which the peptides, after generation in the cytosol by the action of proteases, are transferred from one HSP to another, until they are finally accepted by MHC class I molecules in the ER (endoplasmic reticulum). DC-tumor fusion (right) integrated the Ag processing and presentation machinery from DC and rich tumor peptides from tumor. Through this approach, multiple tumor antigenic peptides, including those yet unidentified, are processed by MHC making fusion cells enriched in a wider repertoire of immunogenic peptides. So we think that there may be much more tumor peptides associated with HSPs in DC-tumor fusion cells compared with that from tumor cells. (TIF) [file pone.0126075.s005.tif]

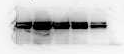

Supplement: S2 Fig — Original figure of Western blot for HSP70 (Lane 1–5: Fc1, Fc2, Tu1, Tu2, positive control); Fig. B. Original figure of Western blot for HSP90 (Lane 1–5: positive control, Fc1, Fc2, Tu1, Tu2); Fig. C. Original figure of Western blot for HSP110 (Lane 1–4: Fc1, Fc2, Tu1, Tu2); Fig. D. Original figure of NC membrane and marker for HSP70 Western blot; Fig. E. Original figure of NC membrane and marker for HSP90 Western blot; Fig. F. Original figure of NC membrane and marker for HSP110 Western blot. (ZIP) [file pone.0126075.s006.zip › FigA.tif]

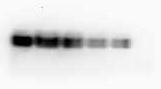

Supplement: S2 Fig — Original figure of Western blot for HSP70 (Lane 1–5: Fc1, Fc2, Tu1, Tu2, positive control); Fig. B. Original figure of Western blot for HSP90 (Lane 1–5: positive control, Fc1, Fc2, Tu1, Tu2); Fig. C. Original figure of Western blot for HSP110 (Lane 1–4: Fc1, Fc2, Tu1, Tu2); Fig. D. Original figure of NC membrane and marker for HSP70 Western blot; Fig. E. Original figure of NC membrane and marker for HSP90 Western blot; Fig. F. Original figure of NC membrane and marker for HSP110 Western blot. (ZIP) [file pone.0126075.s006.zip › FigB.tif]

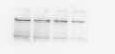

Supplement: S2 Fig — Original figure of Western blot for HSP70 (Lane 1–5: Fc1, Fc2, Tu1, Tu2, positive control); Fig. B. Original figure of Western blot for HSP90 (Lane 1–5: positive control, Fc1, Fc2, Tu1, Tu2); Fig. C. Original figure of Western blot for HSP110 (Lane 1–4: Fc1, Fc2, Tu1, Tu2); Fig. D. Original figure of NC membrane and marker for HSP70 Western blot; Fig. E. Original figure of NC membrane and marker for HSP90 Western blot; Fig. F. Original figure of NC membrane and marker for HSP110 Western blot. (ZIP) [file pone.0126075.s006.zip › FigC.tif]

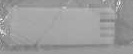

Supplement: S2 Fig — Original figure of Western blot for HSP70 (Lane 1–5: Fc1, Fc2, Tu1, Tu2, positive control); Fig. B. Original figure of Western blot for HSP90 (Lane 1–5: positive control, Fc1, Fc2, Tu1, Tu2); Fig. C. Original figure of Western blot for HSP110 (Lane 1–4: Fc1, Fc2, Tu1, Tu2); Fig. D. Original figure of NC membrane and marker for HSP70 Western blot; Fig. E. Original figure of NC membrane and marker for HSP90 Western blot; Fig. F. Original figure of NC membrane and marker for HSP110 Western blot. (ZIP) [file pone.0126075.s006.zip › FigD.tif]

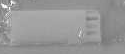

Supplement: S2 Fig — Original figure of Western blot for HSP70 (Lane 1–5: Fc1, Fc2, Tu1, Tu2, positive control); Fig. B. Original figure of Western blot for HSP90 (Lane 1–5: positive control, Fc1, Fc2, Tu1, Tu2); Fig. C. Original figure of Western blot for HSP110 (Lane 1–4: Fc1, Fc2, Tu1, Tu2); Fig. D. Original figure of NC membrane and marker for HSP70 Western blot; Fig. E. Original figure of NC membrane and marker for HSP90 Western blot; Fig. F. Original figure of NC membrane and marker for HSP110 Western blot. (ZIP) [file pone.0126075.s006.zip › FigE.tif]

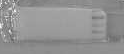

Supplement: S2 Fig — Original figure of Western blot for HSP70 (Lane 1–5: Fc1, Fc2, Tu1, Tu2, positive control); Fig. B. Original figure of Western blot for HSP90 (Lane 1–5: positive control, Fc1, Fc2, Tu1, Tu2); Fig. C. Original figure of Western blot for HSP110 (Lane 1–4: Fc1, Fc2, Tu1, Tu2); Fig. D. Original figure of NC membrane and marker for HSP70 Western blot; Fig. E. Original figure of NC membrane and marker for HSP90 Western blot; Fig. F. Original figure of NC membrane and marker for HSP110 Western blot. (ZIP) [file pone.0126075.s006.zip › FigF.tif]
