# Supplementary material for: Dendritic-Tumor Fusion Cells Derived Heat Shock Protein70-Peptide Complex Has Enhanced Immunogenicity
Source: PLoS One. 2015 May 11;10(5):e0126075. doi: 10.1371/journal.pone.0126075 (PMC4427282; doi:10.1371/journal.pone.0126075)
Supplement: S1 Statistics — (PDF) [file pone.0126075.s007.pdf]

1. CTL for patient1 Oneway

1.1 E/T ratio 12.5:1 against auto breast tumor cells

Descriptives

var

	N	Mean	Std. Deviation	Std. Error	95% Confidence Interval for Mean		Minimum	Maximum
					Lower Bound	Upper Bound		
1.00	3	23.6667	2.08167	1.20185	18.4955	28.8378	22.00	26.00
2.00	3	14.3333	2.08167	1.20185	9.1622	19.5045	12.00	16.00
3.00	3	9.0000	2.00000	1.15470	4.0317	13.9683	7.00	11.00
Total	9	15.6667	6.67083	2.22361	10.5390	20.7943	7.00	26.00

ANOVA

var

	Sum of Squares	df	Mean Square	F	Sig.
Between Groups	330.667	2	165.333	39.158	.000
Within Groups	25.333	6	4.222		
Total	356.000	8			

Post Hoc Tests

Multiple Comparisons

Dependent Variable: var

	(I) group	(J) group	Mean Difference (I-J)	Std. Error	Sig.	95% Confidence Interval	
						Lower Bound	Upper Bound
LSD	1.00	2.00	9.33333(*)	1.67774	.001	5.2280	13.4386
		3.00	14.66667(*)	1.67774	.000	10.5614	18.7720
	2.00	1.00	-9.33333(*)	1.67774	.001	-13.4386	-5.2280
		3.00	5.33333(*)	1.67774	.019	1.2280	9.4386
	3.00	1.00	-14.66667(*)	1.67774	.000	-18.7720	-10.5614
		2.00	-5.33333(*)	1.67774	.019	-9.4386	-1.2280

* The mean difference is significant at the .05 level.

1.2 E/T ratio 25:1 against auto breast tumor cells

Descriptives

var

	N	Mean	Std. Deviation	Std. Error	95% Confidence Interval for Mean		Minimum	Maximum
					Lower Bound	Upper Bound		
1.00	3	39.2667	1.70098	.98206	35.0412	43.4921	37.60	41.00
2.00	3	23.3333	4.04145	2.33333	13.2938	33.3729	19.00	27.00
3.00	3	13.0000	2.64575	1.52753	6.4276	19.5724	11.00	16.00
Total	9	25.2000	11.74223	3.91408	16.1741	34.2259	11.00	41.00

ANOVA

var

	Sum of Squares	df	Mean Square	F	Sig.
Between Groups	1050.587	2	525.293	60.087	.000
Within Groups	52.453	6	8.742		
Total	1103.040	8			

Post Hoc Tests

Multiple Comparisons

Dependent Variable: var

	(I) group	(J) group	Mean Difference (I-J)	Std. Error	Sig.	95% Confidence Interval	
						Lower Bound	Upper Bound
LSD	1.00	2.00	15.93333(*)	2.41416	.001	10.0261	21.8406
		3.00	26.26667(*)	2.41416	.000	20.3594	32.1739
	2.00	1.00	-15.93333(*)	2.41416	.001	-21.8406	-10.0261
		3.00	10.33333(*)	2.41416	.005	4.4261	16.2406
	3.00	1.00	-26.26667(*)	2.41416	.000	-32.1739	-20.3594
		2.00	-10.33333(*)	2.41416	.005	-16.2406	-4.4261

* The mean difference is significant at the .05 level.

1.3 E/T ratio 50:1 against auto breast tumor cells

Descriptives

var

	N	Mean	Std. Deviation	Std. Error	95% Confidence Interval for Mean		Minimum	Maximum
					Lower Bound	Upper Bound		
1.00	3	67.0000	5.00000	2.88675	54.5793	79.4207	62.00	72.00
2.00	3	38.0000	2.00000	1.15470	33.0317	42.9683	36.00	40.00
3.00	3	15.6667	2.88675	1.66667	8.4956	22.8378	14.00	19.00
Total	9	40.2222	22.49877	7.49959	22.9281	57.5163	14.00	72.00

ANOVA

var

	Sum of Squares	df	Mean Square	F	Sig.
Between Groups	3974.889	2	1987.444	159.705	.000
Within Groups	74.667	6	12.444		
Total	4049.556	8			

Post Hoc Tests

Multiple Comparisons

Dependent Variable: var

	(I) group	(J) group	Mean Difference (I-J)	Std. Error	Sig.	95% Confidence Interval	
						Lower Bound	Upper Bound
LSD	1.00	2.00	29.00000(*)	2.88033	.000	21.9521	36.0479
		3.00	51.33333(*)	2.88033	.000	44.2854	58.3812
	2.00	1.00	-29.00000(*)	2.88033	.000	-36.0479	-21.9521
		3.00	22.33333(*)	2.88033	.000	15.2854	29.3812
	3.00	1.00	-51.33333(*)	2.88033	.000	-58.3812	-44.2854
		2.00	-22.33333(*)	2.88033	.000	-29.3812	-15.2854

* The mean difference is significant at the .05 level.

1.4 E/T ratio 50:1 against different tumor target

Descriptives

k562

	N	Mean	Std. Deviation	Std. Error	95% Confidence Interval for Mean		Minimum	Maximum
					Lower Bound	Upper Bound		
1.00	3	67.0000	5.00000	2.88675	54.5793	79.4207	62.00	72.00
2.00	3	40.0000	2.00000	1.15470	35.0317	44.9683	38.00	42.00
3.00	3	12.3333	1.52753	.88192	8.5388	16.1279	11.00	14.00
4.00	3	12.6667	2.51661	1.45297	6.4151	18.9183	10.00	15.00
5.00	3	8.6667	.57735	.33333	7.2324	10.1009	8.00	9.00
6.00	3	8.3333	1.52753	.88192	4.5388	12.1279	7.00	10.00
Total	18	24.8333	22.51601	5.30707	13.6364	36.0303	7.00	72.00

ANOVA

	Sum of Squares	df	Mean Square	F	Sig.

Between Groups	8537.833	5	1707.567	254.018	.000
Within Groups	80.667	12	6.722		
Total	8618.500	17			

Post Hoc Tests

Multiple Comparisons

Dependent Variable: k562

	(I) group	(J) group	Mean Difference (I-J)	Std. Error	Sig.	95% Confidence Interval Lower Bound	95% Confidence Interval Upper Bound
LSD	1.00	2.00	27.00000(*)	2.11695	.000	22.3876	31.6124
		3.00	54.66667(*)	2.11695	.000	50.0542	59.2791
		4.00	54.33333(*)	2.11695	.000	49.7209	58.9458
		5.00	58.33333(*)	2.11695	.000	53.7209	62.9458
		6.00	58.66667(*)	2.11695	.000	54.0542	63.2791
	2.00	1.00	-27.00000(*)	2.11695	.000	-31.6124	-22.3876
		3.00	27.66667(*)	2.11695	.000	23.0542	32.2791
		4.00	27.33333(*)	2.11695	.000	22.7209	31.9458
		5.00	31.33333(*)	2.11695	.000	26.7209	35.9458
		6.00	31.66667(*)	2.11695	.000	27.0542	36.2791
	3.00	1.00	-54.66667(*)	2.11695	.000	-59.2791	-50.0542
		2.00	-27.66667(*)	2.11695	.000	-32.2791	-23.0542
		4.00	-.33333	2.11695	.878	-4.9458	4.2791
		5.00	3.66667	2.11695	.109	-.9458	8.2791
		6.00	4.00000	2.11695	.083	-.6124	8.6124
	4.00	1.00	-54.33333(*)	2.11695	.000	-58.9458	-49.7209
		2.00	-27.33333(*)	2.11695	.000	-31.9458	-22.7209
		3.00	.33333	2.11695	.878	-4.2791	4.9458
		5.00	4.00000	2.11695	.083	-.6124	8.6124
		6.00	4.33333	2.11695	.063	-.2791	8.9458
	5.00	1.00	-58.33333(*)	2.11695	.000	-62.9458	-53.7209
		2.00	-31.33333(*)	2.11695	.000	-35.9458	-26.7209
		3.00	-3.66667	2.11695	.109	-8.2791	.9458
		4.00	-4.00000	2.11695	.083	-8.6124	.6124
		6.00	.33333	2.11695	.878	-4.2791	4.9458
	6.00	1.00	-58.66667(*)	2.11695	.000	-63.2791	-54.0542
		2.00	-31.66667(*)	2.11695	.000	-36.2791	-27.0542
		3.00	-4.00000	2.11695	.083	-8.6124	.6124
		4.00	-4.33333	2.11695	.063	-8.9458	.2791
		5.00	-.33333	2.11695	.878	-4.9458	4.2791

* The mean difference is significant at the .05 level.

1.5 E/T ratio 50:1 against different tumor target by HLA block

Descriptives

block

	N	Mean	Std. Deviation	Std. Error	95% Confidence Interval for Mean		Minimum	Maximum
					Lower Bound	Upper Bound		
1.00	3	10.0000	1.00000	.57735	7.5159	12.4841	9.00	11.00
2.00	3	9.6667	1.15470	.66667	6.7982	12.5351	9.00	11.00
3.00	3	9.6667	1.52753	.88192	5.8721	13.4612	8.00	11.00
4.00	3	10.3333	1.52753	.88192	6.5388	14.1279	9.00	12.00
5.00	3	9.6667	.57735	.33333	8.2324	11.1009	9.00	10.00
6.00	3	9.3333	1.52753	.88192	5.5388	13.1279	8.00	11.00
Total	18	9.7778	1.11437	.26266	9.2236	10.3319	8.00	12.00

ANOVA

block

	Sum of Squares	df	Mean Square	F	Sig.
Between Groups	1.778	5	.356	.221	.947
Within Groups	19.333	12	1.611		
Total	21.111	17			

Post Hoc Tests

Multiple Comparisons

Dependent Variable: block

	(I) group	(J) group	Mean Difference (I-J)	Std. Error	Sig.	95% Confidence Interval	
						Lower Bound	Upper Bound
LSD	1.00	2.00	.33333	1.03638	.753	-1.9247	2.5914
		3.00	.33333	1.03638	.753	-1.9247	2.5914
		4.00	-.33333	1.03638	.753	-2.5914	1.9247
		5.00	.33333	1.03638	.753	-1.9247	2.5914
		6.00	.66667	1.03638	.532	-1.5914	2.9247
	2.00	1.00	-.33333	1.03638	.753	-2.5914	1.9247
		3.00	.00000	1.03638	1.000	-2.2581	2.2581
		4.00	-.66667	1.03638	.532	-2.9247	1.5914
		5.00	.00000	1.03638	1.000	-2.2581	2.2581
		6.00	.33333	1.03638	.753	-1.9247	2.5914
	3.00	1.00	-.33333	1.03638	.753	-2.5914	1.9247
		2.00	.00000	1.03638	1.000	-2.2581	2.2581
		4.00	-.66667	1.03638	.532	-2.9247	1.5914
		5.00	.00000	1.03638	1.000	-2.2581	2.2581
		6.00	.33333	1.03638	.753	-1.9247	2.5914
	4.00	1.00	.33333	1.03638	.753	-1.9247	2.5914

	2.00	.66667	1.03638	.532	-1.5914	2.9247
	3.00	.66667	1.03638	.532	-1.5914	2.9247
	5.00	.66667	1.03638	.532	-1.5914	2.9247
	6.00	1.00000	1.03638	.354	-1.2581	3.2581
5.00	1.00	-.33333	1.03638	.753	-2.5914	1.9247
	2.00	.00000	1.03638	1.000	-2.2581	2.2581
	3.00	.00000	1.03638	1.000	-2.2581	2.2581
	4.00	-.66667	1.03638	.532	-2.9247	1.5914
	6.00	.33333	1.03638	.753	-1.9247	2.5914
6.00	1.00	-.66667	1.03638	.532	-2.9247	1.5914
	2.00	-.33333	1.03638	.753	-2.5914	1.9247
	3.00	-.33333	1.03638	.753	-2.5914	1.9247
	4.00	-1.00000	1.03638	.354	-3.2581	1.2581
	5.00	-.33333	1.03638	.753	-2.5914	1.9247

2. CTL for patient2 Oneway

2.1 E/T ratio 12.5:1 against auto breast tumor cells

Descriptives

var

	N	Mean	Std. Deviation	Std. Error	95% Confidence Interval for Mean		Minimum	Maximum
					Lower Bound	Upper Bound		
1.00	3	26.6667	2.08167	1.20185	21.4955	31.8378	25.00	29.00
2.00	3	15.3333	3.78594	2.18581	5.9285	24.7381	11.00	18.00
3.00	3	10.6667	2.08167	1.20185	5.4955	15.8378	9.00	13.00
Total	9	17.5556	7.51850	2.50617	11.7763	23.3348	9.00	29.00

ANOVA

var

	Sum of Squares	df	Mean Square	F	Sig.
Between Groups	406.222	2	203.111	26.493	.001
Within Groups	46.000	6	7.667		
Total	452.222	8			

Post Hoc Tests

Multiple Comparisons

Dependent Variable: var
LSD

(I) group	(J) group	Mean Difference (I-J)	Std. Error	Sig.	95% Confidence Interval

					Lower Bound	Upper Bound
1.00	2.00	11.33333(*)	2.26078	.002	5.8014	16.8653
	3.00	16.00000(*)	2.26078	.000	10.4681	21.5319
2.00	1.00	-11.33333(*)	2.26078	.002	-16.8653	-5.8014
	3.00	4.66667	2.26078	.085	-.8653	10.1986
3.00	1.00	-16.00000(*)	2.26078	.000	-21.5319	-10.4681
	2.00	-4.66667	2.26078	.085	-10.1986	.8653

* The mean difference is significant at the .05 level.

2.2 E/T ratio 25:1 against auto breast tumor cells

Descriptives

var

	N	Mean	Std. Deviation	Std. Error	95% Confidence Interval for Mean		Minimum	Maximum
					Lower Bound	Upper Bound		
1.00	3	41.2333	1.76163	1.01708	36.8572	45.6095	39.60	43.10
2.00	3	26.3333	2.51661	1.45297	20.0817	32.5849	24.00	29.00
3.00	3	14.3333	3.21455	1.85592	6.3479	22.3187	12.00	18.00
Total	9	27.3000	11.88045	3.96015	18.1679	36.4321	12.00	43.10

ANOVA

var

	Sum of Squares	df	Mean Square	F	Sig.
Between Groups	1089.620	2	544.810	82.672	.000
Within Groups	39.540	6	6.590		
Total	1129.160	8			

Post Hoc Tests

Multiple Comparisons

Dependent Variable: var
LSD

(I) group	(J) group	Mean Difference (I-J)	Std. Error	Sig.	95% Confidence Interval	
					Lower Bound	Upper Bound
1.00	2.00	14.90000(*)	2.09603	.000	9.7712	20.0288
	3.00	26.90000(*)	2.09603	.000	21.7712	32.0288
2.00	1.00	-14.90000(*)	2.09603	.000	-20.0288	-9.7712
	3.00	12.00000(*)	2.09603	.001	6.8712	17.1288
3.00	1.00	-26.90000(*)	2.09603	.000	-32.0288	-21.7712
	2.00	-12.00000(*)	2.09603	.001	-17.1288	-6.8712

* The mean difference is significant at the .05 level.

2.3 E/T ratio 50:1 against auto breast tumor cells

Descriptives

var

	N	Mean	Std. Deviation	Std. Error	95% Confidence Interval for Mean		Minimum	Maximum
					Lower Bound	Upper Bound		
1.00	3	72.0000	5.00000	2.88675	59.5793	84.4207	67.00	77.00
2.00	3	40.3333	3.78594	2.18581	30.9285	49.7381	36.00	43.00
3.00	3	18.6667	3.78594	2.18581	9.2619	28.0715	16.00	23.00
Total	9	43.6667	23.51595	7.83865	25.5907	61.7426	16.00	77.00

ANOVA

var

	Sum of Squares	df	Mean Square	F	Sig.
Between Groups	4316.667	2	2158.333	120.652	.000
Within Groups	107.333	6	17.889		
Total	4424.000	8			

Post Hoc Tests

Multiple Comparisons

Dependent Variable: var
LSD

(I) group	(J) group	Mean Difference (I-J)	Std. Error	Sig.	95% Confidence Interval	
					Lower Bound	Upper Bound
1.00	2.00	31.66667(*)	3.45339	.000	23.2165	40.1168
	3.00	53.33333(*)	3.45339	.000	44.8832	61.7835
2.00	1.00	-31.66667(*)	3.45339	.000	-40.1168	-23.2165
	3.00	21.66667(*)	3.45339	.001	13.2165	30.1168
3.00	1.00	-53.33333(*)	3.45339	.000	-61.7835	-44.8832
	2.00	-21.66667(*)	3.45339	.001	-30.1168	-13.2165

* The mean difference is significant at the .05 level.

2.4 E/T ratio 50:1 against different tumor target

Descriptives

k562

	N	Mean	Std. Deviation	Std. Error	95% Confidence Interval for Mean Lower Bound	95% Confidence Interval for Mean Upper Bound	Minimum	Maximum
1.00	3	72.0000	5.00000	2.88675	59.5793	84.4207	67.00	77.00
2.00	3	46.6667	1.15470	.66667	43.7982	49.5351	46.00	48.00
3.00	3	13.6667	1.52753	.88192	9.8721	17.4612	12.00	15.00
4.00	3	12.6667	.57735	.33333	11.2324	14.1009	12.00	13.00
5.00	3	11.6667	.57735	.33333	10.2324	13.1009	11.00	12.00
6.00	3	10.3333	.57735	.33333	8.8991	11.7676	10.00	11.00
Total	18	27.8333	24.21776	5.70818	15.7901	39.8765	10.00	77.00

ANOVA

k562

	Sum of Squares	df	Mean Square	F	Sig.
Between Groups	9911.167	5	1982.233	400.901	.000
Within Groups	59.333	12	4.944		
Total	9970.500	17			

Post Hoc Tests

Multiple Comparisons

Dependent Variable: k562
LSD

(I) group	(J) group	Mean Difference (I-J)	Std. Error	Sig.	95% Confidence Interval Lower Bound	95% Confidence Interval Upper Bound
1.00	2.00	25.33333(*)	1.81557	.000	21.3775	29.2891
	3.00	58.33333(*)	1.81557	.000	54.3775	62.2891
	4.00	59.33333(*)	1.81557	.000	55.3775	63.2891
	5.00	60.33333(*)	1.81557	.000	56.3775	64.2891
	6.00	61.66667(*)	1.81557	.000	57.7109	65.6225
2.00	1.00	-25.33333(*)	1.81557	.000	-29.2891	-21.3775
	3.00	33.00000(*)	1.81557	.000	29.0442	36.9558
	4.00	34.00000(*)	1.81557	.000	30.0442	37.9558
	5.00	35.00000(*)	1.81557	.000	31.0442	38.9558
	6.00	36.33333(*)	1.81557	.000	32.3775	40.2891
3.00	1.00	-58.33333(*)	1.81557	.000	-62.2891	-54.3775
	2.00	-33.00000(*)	1.81557	.000	-36.9558	-29.0442
	4.00	1.00000	1.81557	.592	-2.9558	4.9558
	5.00	2.00000	1.81557	.292	-1.9558	5.9558
	6.00	3.33333	1.81557	.091	-.6225	7.2891
4.00	1.00	-59.33333(*)	1.81557	.000	-63.2891	-55.3775

	2.00	-34.00000(*)	1.81557	.000	-37.9558	-30.0442
	3.00	-1.00000	1.81557	.592	-4.9558	2.9558
	5.00	1.00000	1.81557	.592	-2.9558	4.9558
	6.00	2.33333	1.81557	.223	-1.6225	6.2891
5.00	1.00	-60.33333(*)	1.81557	.000	-64.2891	-56.3775
	2.00	-35.00000(*)	1.81557	.000	-38.9558	-31.0442
	3.00	-2.00000	1.81557	.292	-5.9558	1.9558
	4.00	-1.00000	1.81557	.592	-4.9558	2.9558
	6.00	1.33333	1.81557	.477	-2.6225	5.2891
6.00	1.00	-61.66667(*)	1.81557	.000	-65.6225	-57.7109
	2.00	-36.33333(*)	1.81557	.000	-40.2891	-32.3775
	3.00	-3.33333	1.81557	.091	-7.2891	.6225
	4.00	-2.33333	1.81557	.223	-6.2891	1.6225
	5.00	-1.33333	1.81557	.477	-5.2891	2.6225

* The mean difference is significant at the .05 level.

2.5 E/T ratio 50:1 against different tumor target by HLA block

Descriptives

block

	N	Mean	Std. Deviation	Std. Error	95% Confidence Interval for Mean		Minimum	Maximum
					Lower Bound	Upper Bound		
1.00	3	9.0000	1.00000	.57735	6.5159	11.4841	8.00	10.00
2.00	3	8.3333	.57735	.33333	6.8991	9.7676	8.00	9.00
3.00	3	9.3333	1.52753	.88192	5.5388	13.1279	8.00	11.00
4.00	3	8.3333	1.52753	.88192	4.5388	12.1279	7.00	10.00
5.00	3	8.0000	1.00000	.57735	5.5159	10.4841	7.00	9.00
6.00	3	9.0000	1.00000	.57735	6.5159	11.4841	8.00	10.00
Total	18	8.6667	1.08465	.25565	8.1273	9.2061	7.00	11.00

ANOVA

block

	Sum of Squares	df	Mean Square	F	Sig.
Between Groups	4.000	5	.800	.600	.701
Within Groups	16.000	12	1.333		
Total	20.000	17			

Post Hoc Tests

Multiple Comparisons

Dependent Variable: block

LSD

(I) group	(J) group	Mean Difference (I-J)	Std. Error	Sig.	95% Confidence Interval	
					Lower Bound	Upper Bound
1.00	2.00	.66667	.94281	.493	-1.3875	2.7209
	3.00	-.33333	.94281	.730	-2.3875	1.7209
	4.00	.66667	.94281	.493	-1.3875	2.7209
	5.00	1.00000	.94281	.310	-1.0542	3.0542
	6.00	.00000	.94281	1.000	-2.0542	2.0542
2.00	1.00	-.66667	.94281	.493	-2.7209	1.3875
	3.00	-1.00000	.94281	.310	-3.0542	1.0542
	4.00	.00000	.94281	1.000	-2.0542	2.0542
	5.00	.33333	.94281	.730	-1.7209	2.3875
	6.00	-.66667	.94281	.493	-2.7209	1.3875
3.00	1.00	.33333	.94281	.730	-1.7209	2.3875
	2.00	1.00000	.94281	.310	-1.0542	3.0542
	4.00	1.00000	.94281	.310	-1.0542	3.0542
	5.00	1.33333	.94281	.183	-.7209	3.3875
	6.00	.33333	.94281	.730	-1.7209	2.3875
4.00	1.00	-.66667	.94281	.493	-2.7209	1.3875
	2.00	.00000	.94281	1.000	-2.0542	2.0542
	3.00	-1.00000	.94281	.310	-3.0542	1.0542
	5.00	.33333	.94281	.730	-1.7209	2.3875
	6.00	-.66667	.94281	.493	-2.7209	1.3875
5.00	1.00	-1.00000	.94281	.310	-3.0542	1.0542
	2.00	-.33333	.94281	.730	-2.3875	1.7209
	3.00	-1.33333	.94281	.183	-3.3875	.7209
	4.00	-.33333	.94281	.730	-2.3875	1.7209
	6.00	-1.00000	.94281	.310	-3.0542	1.0542
6.00	1.00	.00000	.94281	1.000	-2.0542	2.0542
	2.00	.66667	.94281	.493	-1.3875	2.7209
	3.00	-.33333	.94281	.730	-2.3875	1.7209
	4.00	.66667	.94281	.493	-1.3875	2.7209
	5.00	1.00000	.94281	.310	-1.0542	3.0542

3. CTL for patient3 Oneway

3.1 E/T ratio 12.5:1 against auto breast tumor cells

Descriptives

var

	N	Mean	Std. Deviation	Std. Error	95% Confidence Interval for Mean		Minimum	Maximum
					Lower Bound	Upper Bound		

1.00	3	22.3333	2.30940	1.33333	16.5965	28.0702	21.00	25.00
2.00	3	13.6667	1.52753	.88192	9.8721	17.4612	12.00	15.00
3.00	3	8.0000	1.00000	.57735	5.5159	10.4841	7.00	9.00
Total	9	14.6667	6.42262	2.14087	9.7298	19.6035	7.00	25.00

ANOVA

var

	Sum of Squares	df	Mean Square	F	Sig.
Between Groups	312.667	2	156.333	54.115	.000
Within Groups	17.333	6	2.889		
Total	330.000	8			

Post Hoc Tests

Multiple Comparisons

Dependent Variable: var
LSD

(I) group	(J) group	Mean Difference (I-J)	Std. Error	Sig.	95% Confidence Interval	
					Lower Bound	Upper Bound
1.00	2.00	8.66667(*)	1.38778	.001	5.2709	12.0624
	3.00	14.33333(*)	1.38778	.000	10.9376	17.7291
2.00	1.00	-8.66667(*)	1.38778	.001	-12.0624	-5.2709
	3.00	5.66667(*)	1.38778	.006	2.2709	9.0624
3.00	1.00	-14.33333(*)	1.38778	.000	-17.7291	-10.9376
	2.00	-5.66667(*)	1.38778	.006	-9.0624	-2.2709

* The mean difference is significant at the .05 level.

3.2 E/T ratio 25:1 against auto breast tumor cells

Descriptives

var

	N	Mean	Std. Deviation	Std. Error	95% Confidence Interval for Mean		Minimum	Maximum
					Lower Bound	Upper Bound		
1.00	3	36.2333	2.25019	1.29915	30.6436	41.8231	34.00	38.50
2.00	3	22.0000	2.00000	1.15470	17.0317	26.9683	20.00	24.00
3.00	3	12.0000	2.64575	1.52753	5.4276	18.5724	10.00	15.00
Total	9	23.4111	10.73527	3.57842	15.1592	31.6630	10.00	38.50

ANOVA

var

	Sum of Squares	df	Mean Square	F	Sig.
Between Groups	889.842	2	444.921	83.094	.000
Within Groups	32.127	6	5.354		
Total	921.969	8			

Post Hoc Tests

Multiple Comparisons

Dependent Variable: var
LSD

(I) group	(J) group	Mean Difference (I-J)	Std. Error	Sig.	95% Confidence Interval	
					Lower Bound	Upper Bound
1.00	2.00	14.23333(*)	1.88935	.000	9.6103	18.8564
	3.00	24.23333(*)	1.88935	.000	19.6103	28.8564
2.00	1.00	-14.23333(*)	1.88935	.000	-18.8564	-9.6103
	3.00	10.00000(*)	1.88935	.002	5.3769	14.6231
3.00	1.00	-24.23333(*)	1.88935	.000	-28.8564	-19.6103
	2.00	-10.00000(*)	1.88935	.002	-14.6231	-5.3769

* The mean difference is significant at the .05 level.

3.3 E/T ratio 50:1 against auto breast tumor cells

Descriptives

var

	N	Mean	Std. Deviation	Std. Error	95% Confidence Interval for Mean		Minimum	Maximum
					Lower Bound	Upper Bound		
1.00	3	62.0000	5.00000	2.88675	49.5793	74.4207	57.00	67.00
2.00	3	33.6667	1.52753	.88192	29.8721	37.4612	32.00	35.00
3.00	3	11.6667	2.88675	1.66667	4.4956	18.8378	10.00	15.00
Total	9	35.7778	22.05549	7.35183	18.8244	52.7311	10.00	67.00

ANOVA

var

	Sum of Squares	df	Mean Square	F	Sig.
Between Groups	3820.222	2	1910.111	160.664	.000
Within Groups	71.333	6	11.889		
Total	3891.556	8			

Post Hoc Tests

Multiple Comparisons

Dependent Variable: var
LSD

(I) group	(J) group	Mean Difference (I-J)	Std. Error	Sig.	95% Confidence Interval Lower Bound	95% Confidence Interval Upper Bound
1.00	2.00	28.33333(*)	2.81530	.000	21.4445	35.2221
	3.00	50.33333(*)	2.81530	.000	43.4445	57.2221
2.00	1.00	-28.33333(*)	2.81530	.000	-35.2221	-21.4445
	3.00	22.00000(*)	2.81530	.000	15.1112	28.8888
3.00	1.00	-50.33333(*)	2.81530	.000	-57.2221	-43.4445
	2.00	-22.00000(*)	2.81530	.000	-28.8888	-15.1112

* The mean difference is significant at the .05 level.

3.4 E/T ratio 50:1 against different tumor target

Descriptives

k562

	N	Mean	Std. Deviation	Std. Error	95% Confidence Interval for Mean Lower Bound	95% Confidence Interval for Mean Upper Bound	Minimum	Maximum
1.00	3	62.0000	5.00000	2.88675	49.5793	74.4207	57.00	67.00
2.00	3	12.3333	2.51661	1.45297	6.0817	18.5849	10.00	15.00
3.00	3	42.3333	5.85947	3.38296	27.7776	56.8891	38.00	49.00
4.00	3	14.0000	1.00000	.57735	11.5159	16.4841	13.00	15.00
5.00	3	12.6667	2.08167	1.20185	7.4955	17.8378	11.00	15.00
6.00	3	13.3333	.57735	.33333	11.8991	14.7676	13.00	14.00
Total	18	26.1111	20.05548	4.72712	16.1378	36.0845	10.00	67.00

ANOVA

k562

	Sum of Squares	df	Mean Square	F	Sig.
Between Groups	6695.111	5	1339.022	112.628	.000
Within Groups	142.667	12	11.889		
Total	6837.778	17			

Post Hoc Tests

Multiple Comparisons

Dependent Variable: k562
LSD

(I) group	(J) group	Mean Difference (I-J)	Std. Error	Sig.	95% Confidence Interval Lower Bound	95% Confidence Interval Upper Bound
1.00	2.00	49.66667(*)	2.81530	.000	43.5327	55.8007
	3.00	19.66667(*)	2.81530	.000	13.5327	25.8007
	4.00	48.00000(*)	2.81530	.000	41.8660	54.1340
	5.00	49.33333(*)	2.81530	.000	43.1993	55.4673
	6.00	48.66667(*)	2.81530	.000	42.5327	54.8007
2.00	1.00	-49.66667(*)	2.81530	.000	-55.8007	-43.5327
	3.00	-30.00000(*)	2.81530	.000	-36.1340	-23.8660
	4.00	-1.66667	2.81530	.565	-7.8007	4.4673
	5.00	-.33333	2.81530	.908	-6.4673	5.8007
	6.00	-1.00000	2.81530	.729	-7.1340	5.1340
3.00	1.00	-19.66667(*)	2.81530	.000	-25.8007	-13.5327
	2.00	30.00000(*)	2.81530	.000	23.8660	36.1340
	4.00	28.33333(*)	2.81530	.000	22.1993	34.4673
	5.00	29.66667(*)	2.81530	.000	23.5327	35.8007
	6.00	29.00000(*)	2.81530	.000	22.8660	35.1340
4.00	1.00	-48.00000(*)	2.81530	.000	-54.1340	-41.8660
	2.00	1.66667	2.81530	.565	-4.4673	7.8007
	3.00	-28.33333(*)	2.81530	.000	-34.4673	-22.1993
	5.00	1.33333	2.81530	.644	-4.8007	7.4673
	6.00	.66667	2.81530	.817	-5.4673	6.8007
5.00	1.00	-49.33333(*)	2.81530	.000	-55.4673	-43.1993
	2.00	.33333	2.81530	.908	-5.8007	6.4673
	3.00	-29.66667(*)	2.81530	.000	-35.8007	-23.5327
	4.00	-1.33333	2.81530	.644	-7.4673	4.8007
	6.00	-.66667	2.81530	.817	-6.8007	5.4673
6.00	1.00	-48.66667(*)	2.81530	.000	-54.8007	-42.5327
	2.00	1.00000	2.81530	.729	-5.1340	7.1340
	3.00	-29.00000(*)	2.81530	.000	-35.1340	-22.8660
	4.00	-.66667	2.81530	.817	-6.8007	5.4673
	5.00	.66667	2.81530	.817	-5.4673	6.8007

* The mean difference is significant at the .05 level.

3.5 E/T ratio 50:1 against different tumor target by HLA block

Descriptives

block

	N	Mean	Std. Deviation	Std. Error	95% Confidence Interval for Mean Lower Bound	95% Confidence Interval for Mean Upper Bound	Minimum	Maximum

1.00	3	10.0000	1.73205	1.00000	5.6973	14.3027	8.00	11.00
2.00	3	9.3333	.57735	.33333	7.8991	10.7676	9.00	10.00
3.00	3	8.3333	1.15470	.66667	5.4649	11.2018	7.00	9.00
4.00	3	10.6667	1.15470	.66667	7.7982	13.5351	10.00	12.00
5.00	3	8.6667	.57735	.33333	7.2324	10.1009	8.00	9.00
6.00	3	10.3333	1.15470	.66667	7.4649	13.2018	9.00	11.00
Total	18	9.5556	1.29352	.30489	8.9123	10.1988	7.00	12.00

ANOVA

block

	Sum of Squares	df	Mean Square	F	Sig.
Between Groups	13.111	5	2.622	2.052	.143
Within Groups	15.333	12	1.278		
Total	28.444	17			

Post Hoc Tests

Multiple Comparisons

Dependent Variable: block
LSD

(I) group	(J) group	Mean Difference (I-J)	Std. Error	Sig.	95% Confidence Interval	
					Lower Bound	Upper Bound
1.00	2.00	.66667	.92296	.484	-1.3443	2.6776
	3.00	1.66667	.92296	.096	-.3443	3.6776
	4.00	-.66667	.92296	.484	-2.6776	1.3443
	5.00	1.33333	.92296	.174	-.6776	3.3443
	6.00	-.33333	.92296	.724	-2.3443	1.6776
2.00	1.00	-.66667	.92296	.484	-2.6776	1.3443
	3.00	1.00000	.92296	.300	-1.0110	3.0110
	4.00	-1.33333	.92296	.174	-3.3443	.6776
	5.00	.66667	.92296	.484	-1.3443	2.6776
	6.00	-1.00000	.92296	.300	-3.0110	1.0110
3.00	1.00	-1.66667	.92296	.096	-3.6776	.3443
	2.00	-1.00000	.92296	.300	-3.0110	1.0110
	4.00	-2.33333(*)	.92296	.027	-4.3443	-.3224
	5.00	-.33333	.92296	.724	-2.3443	1.6776
	6.00	-2.00000	.92296	.051	-4.0110	.0110
4.00	1.00	.66667	.92296	.484	-1.3443	2.6776
	2.00	1.33333	.92296	.174	-.6776	3.3443
	3.00	2.33333(*)	.92296	.027	.3224	4.3443
	5.00	2.00000	.92296	.051	-.0110	4.0110
	6.00	.33333	.92296	.724	-1.6776	2.3443

5.00	1.00	-1.33333	.92296	.174	-3.3443	.6776
	2.00	-.66667	.92296	.484	-2.6776	1.3443
	3.00	.33333	.92296	.724	-1.6776	2.3443
	4.00	-2.00000	.92296	.051	-4.0110	.0110
	6.00	-1.66667	.92296	.096	-3.6776	.3443
6.00	1.00	.33333	.92296	.724	-1.6776	2.3443
	2.00	1.00000	.92296	.300	-1.0110	3.0110
	3.00	2.00000	.92296	.051	-.0110	4.0110
	4.00	-.33333	.92296	.724	-2.3443	1.6776
	5.00	1.66667	.92296	.096	-.3443	3.6776

* The mean difference is significant at the .05 level.

4. CTL for patient4 Oneway

CTL against auto breast tumor cells at ratio 50:1

Descriptives

var

	N	Mean	Std. Deviation	Std. Error	95% Confidence Interval for Mean		Minimum	Maximum
					Lower Bound	Upper Bound		
1.00	3	68.3333	2.08167	1.20185	63.1622	73.5045	66.00	70.00
2.00	3	40.3333	1.52753	.88192	36.5388	44.1279	39.00	42.00
3.00	3	21.6667	2.51661	1.45297	15.4151	27.9183	19.00	24.00
4.00	3	14.6667	1.15470	.66667	11.7982	17.5351	14.00	16.00
Total	12	36.2500	21.74700	6.27782	22.4326	50.0674	14.00	70.00

ANOVA

var

	Sum of Squares	df	Mean Square	F	Sig.
Between Groups	5173.583	3	1724.528	481.264	.000
Within Groups	28.667	8	3.583		
Total	5202.250	11			

Post Hoc Tests

Multiple Comparisons

Dependent Variable: var

	(I) group	(J) group	Mean Difference (I-J)	Std. Error	Sig.	95% Confidence Interval	
						Lower Bound	Upper Bound

LSD	1.00	2.00	28.00000(*)	1.54560	.000	24.4358	31.5642
		3.00	46.66667(*)	1.54560	.000	43.1025	50.2308
		4.00	53.66667(*)	1.54560	.000	50.1025	57.2308
	2.00	1.00	-28.00000(*)	1.54560	.000	-31.5642	-24.4358
		3.00	18.66667(*)	1.54560	.000	15.1025	22.2308
		4.00	25.66667(*)	1.54560	.000	22.1025	29.2308
	3.00	1.00	-46.66667(*)	1.54560	.000	-50.2308	-43.1025
		2.00	-18.66667(*)	1.54560	.000	-22.2308	-15.1025
		4.00	7.00000(*)	1.54560	.002	3.4358	10.5642
	4.00	1.00	-53.66667(*)	1.54560	.000	-57.2308	-50.1025
		2.00	-25.66667(*)	1.54560	.000	-29.2308	-22.1025
		3.00	-7.00000(*)	1.54560	.002	-10.5642	-3.4358

* The mean difference is significant at the .05 level.

5. CTL for patient5 Oneway

CTL against auto breast tumor cells at ratio 50:1

Descriptives

var

	N	Mean	Std. Deviation	Std. Error	95% Confidence Interval for Mean		Minimum	Maximum
					Lower Bound	Upper Bound		
1.00	3	68.6667	2.51661	1.45297	62.4151	74.9183	66.00	71.00
2.00	3	38.3333	1.15470	.66667	35.4649	41.2018	37.00	39.00
3.00	3	18.0000	1.00000	.57735	15.5159	20.4841	17.00	19.00
4.00	3	14.0000	1.00000	.57735	11.5159	16.4841	13.00	15.00
Total	12	34.7500	22.64800	6.53791	20.3601	49.1399	13.00	71.00

ANOVA

var

	Sum of Squares	df	Mean Square	F	Sig.
Between Groups	5622.917	3	1874.306	775.575	.000
Within Groups	19.333	8	2.417		
Total	5642.250	11			

Post Hoc Tests

Multiple Comparisons

Dependent Variable: var
LSD

(I) group	(J) group	Mean Difference (I-J)	Std. Error	Sig.	95% Confidence Interval

						Lower Bound	Upper Bound
1.00	2.00	30.33333(*)	1.26930	.000	27.4063	33.2603	
	3.00	50.66667(*)	1.26930	.000	47.7397	53.5937	
	4.00	54.66667(*)	1.26930	.000	51.7397	57.5937	
2.00	1.00	-30.33333(*)	1.26930	.000	-33.2603	-27.4063	
	3.00	20.33333(*)	1.26930	.000	17.4063	23.2603	
	4.00	24.33333(*)	1.26930	.000	21.4063	27.2603	
3.00	1.00	-50.66667(*)	1.26930	.000	-53.5937	-47.7397	
	2.00	-20.33333(*)	1.26930	.000	-23.2603	-17.4063	
	4.00	4.00000(*)	1.26930	.014	1.0730	6.9270	
4.00	1.00	-54.66667(*)	1.26930	.000	-57.5937	-51.7397	
	2.00	-24.33333(*)	1.26930	.000	-27.2603	-21.4063	
	3.00	-4.00000(*)	1.26930	.014	-6.9270	-1.0730	

* The mean difference is significant at the .05 level.
