# Supplementary material for: Dendritic-Tumor Fusion Cells Derived Heat Shock Protein70-Peptide Complex Has Enhanced Immunogenicity
Source: PLoS One. 2015 May 11;10(5):e0126075. doi: 10.1371/journal.pone.0126075 (PMC4427282; doi:10.1371/journal.pone.0126075)
Supplement: S2 Statistics — (PDF) [file pone.0126075.s008.pdf]

1. IFN-gamma Elispot Oneway for patient1

Descriptives

data

	N	Mean	Std. Deviation	Std. Error	95% Confidence Interval for Mean		Minimum	Maximum
					Lower Bound	Upper Bound		
1.00	3	283.0000	6.55744	3.78594	266.7104	299.2896	277.00	290.00
2.00	3	89.3333	7.02377	4.05518	71.8853	106.7813	82.00	96.00
3.00	3	24.6667	3.05505	1.76383	17.0775	32.2558	22.00	28.00
Total	9	132.3333	116.52682	38.84227	42.7629	221.9038	22.00	290.00

ANOVA

data

	Sum of Squares	df	Mean Square	F	Sig.
Between Groups	108424.667	2	54212.333	1599.708	.000
Within Groups	203.333	6	33.889		
Total	108628.000	8			

Post Hoc Tests

Multiple Comparisons

Dependent Variable: data

	(I) group	(J) group	Mean Difference (I-J)	Std. Error	Sig.	95% Confidence Interval	
						Lower Bound	Upper Bound
LSD	1.00	2.00	193.66667(*)	4.75317	.000	182.0361	205.2972
		3.00	258.33333(*)	4.75317	.000	246.7028	269.9639
	2.00	1.00	-193.66667(*)	4.75317	.000	-205.2972	-182.0361
		3.00	64.66667(*)	4.75317	.000	53.0361	76.2972
	3.00	1.00	-258.33333(*)	4.75317	.000	-269.9639	-246.7028
		2.00	-64.66667(*)	4.75317	.000	-76.2972	-53.0361

* The mean difference is significant at the .05 level.

2. IFN-gamma Elispot Oneway for patient2

Descriptives

data

	N	Mean	Std. Deviation	Std. Error	95% Confidence Interval for Mean		Minimum	Maximum
					Lower Bound	Upper Bound		
1.00	3	325.0000	4.35890	2.51661	314.1719	335.8281	322.00	330.00
2.00	3	164.3333	6.65833	3.84419	147.7931	180.8735	157.00	170.00
3.00	3	49.0000	5.29150	3.05505	35.8552	62.1448	45.00	55.00
Total	9	179.4444	120.14274	40.04758	87.0946	271.7943	45.00	330.00

ANOVA

data

	Sum of Squares	df	Mean Square	F	Sig.
Between Groups	115291.556	2	57645.778	1893.474	.000
Within Groups	182.667	6	30.444		
Total	115474.222	8			

Post Hoc Tests

Multiple Comparisons

Dependent Variable: data
LSD

(I) group	(J) group	Mean Difference (I-J)	Std. Error	Sig.	95% Confidence Interval	
					Lower Bound	Upper Bound
1.00	2.00	160.66667(*)	4.50514	.000	149.6430	171.6903
	3.00	276.00000(*)	4.50514	.000	264.9763	287.0237
2.00	1.00	-160.66667(*)	4.50514	.000	-171.6903	-149.6430
	3.00	115.33333(*)	4.50514	.000	104.3097	126.3570
3.00	1.00	-276.00000(*)	4.50514	.000	-287.0237	-264.9763
	2.00	-115.33333(*)	4.50514	.000	-126.3570	-104.3097

* The mean difference is significant at the .05 level.

3. IFN-gamma Elispot Oneway for patient3

Descriptives

data

	N	Mean	Std. Deviation	Std. Error	95% Confidence Interval for Mean Lower Bound	95% Confidence Interval for Mean Upper Bound	Minimum	Maximum
1.00	3	213.0000	6.08276	3.51188	197.8896	228.1104	209.00	220.00
2.00	3	102.0000	7.21110	4.16333	84.0866	119.9134	96.00	110.00
3.00	3	23.0000	2.64575	1.52753	16.4276	29.5724	21.00	26.00
Total	9	112.6667	82.80549	27.60183	49.0167	176.3166	21.00	220.00

ANOVA

data

	Sum of Squares	df	Mean Square	F	Sig.
Between Groups	54662.000	2	27331.000	854.094	.000
Within Groups	192.000	6	32.000		
Total	54854.000	8			

Post Hoc Tests

Multiple Comparisons

Dependent Variable: data
LSD

(I) group	(J) group	Mean Difference (I-J)	Std. Error	Sig.	95% Confidence Interval Lower Bound	95% Confidence Interval Upper Bound
1.00	2.00	111.00000(*)	4.61880	.000	99.6982	122.3018
	3.00	190.00000(*)	4.61880	.000	178.6982	201.3018
2.00	1.00	-111.00000(*)	4.61880	.000	-122.3018	-99.6982
	3.00	79.00000(*)	4.61880	.000	67.6982	90.3018
3.00	1.00	-190.00000(*)	4.61880	.000	-201.3018	-178.6982
	2.00	-79.00000(*)	4.61880	.000	-90.3018	-67.6982

* The mean difference is significant at the .05 level.
