# Supplementary material for: Dendritic-Tumor Fusion Cells Derived Heat Shock Protein70-Peptide Complex Has Enhanced Immunogenicity
Source: PLoS One. 2015 May 11;10(5):e0126075. doi: 10.1371/journal.pone.0126075 (PMC4427282; doi:10.1371/journal.pone.0126075)
Supplement: S3 Statistics — (PDF) [file pone.0126075.s009.pdf]

1. IFN release by CD4 T cells Oneway

Descriptives

data

	N	Mean	Std. Deviation	Std. Error	95% Confidence Interval for Mean		Minimum	Maximum
					Lower Bound	Upper Bound		
1.00	3	.5533	.40808	.23561	-.4604	1.5671	.20	1.00
2.00	3	1.2933	.53304	.30775	-.0308	2.6175	.90	1.90
3.00	3	6.7233	.63689	.36771	5.1412	8.3055	6.00	7.20
Total	9	2.8567	2.95411	.98470	.5859	5.1274	.20	7.20

ANOVA

data

	Sum of Squares	df	Mean Square	F	Sig.
Between Groups	68.101	2	34.051	119.295	.000
Within Groups	1.713	6	.285		
Total	69.814	8			

Post Hoc Tests

Multiple Comparisons

Dependent Variable: data

	(I) group	(J) group	Mean Difference (I-J)	Std. Error	Sig.	95% Confidence Interval	
						Lower Bound	Upper Bound
LSD	1.00	2.00	-.74000	.43622	.141	-1.8074	.3274
		3.00	-6.17000(*)	.43622	.000	-7.2374	-5.1026
	2.00	1.00	.74000	.43622	.141	-.3274	1.8074
		3.00	-5.43000(*)	.43622	.000	-6.4974	-4.3626
	3.00	1.00	6.17000(*)	.43622	.000	5.1026	7.2374
		2.00	5.43000(*)	.43622	.000	4.3626	6.4974

* The mean difference is significant at the .05 level.

2. IFN release by CD8 T cells Oneway

Descriptives

data

	N	Mean	Std. Deviation	Std. Error	95% Confidence Interval for Mean	Minimum	Maximum

					Lower Bound	Upper Bound		
1.00	3	2.0067	.41004	.23674	.9881	3.0253	1.60	2.42
2.00	3	7.1533	.80407	.46423	5.1559	9.1508	6.40	8.00
3.00	3	15.0000	.91652	.52915	12.7233	17.2767	14.00	15.80
Total	9	8.0533	5.70301	1.90100	3.6696	12.4371	1.60	15.80

ANOVA

data

	Sum of Squares	df	Mean Square	F	Sig.
Between Groups	256.885	2	128.443	232.873	.000
Within Groups	3.309	6	.552		
Total	260.194	8			

Post Hoc Tests

Multiple Comparisons

Dependent Variable: data

	(I) group	(J) group	Mean Difference (I-J)	Std. Error	Sig.	95% Confidence Interval	
						Lower Bound	Upper Bound
LSD	1.00	2.00	-5.14667(*)	.60639	.000	-6.6304	-3.6629
		3.00	-12.99333(*)	.60639	.000	-14.4771	-11.5096
	2.00	1.00	5.14667(*)	.60639	.000	3.6629	6.6304
		3.00	-7.84667(*)	.60639	.000	-9.3304	-6.3629
	3.00	1.00	12.99333(*)	.60639	.000	11.5096	14.4771
		2.00	7.84667(*)	.60639	.000	6.3629	9.3304

* The mean difference is significant at the .05 level.
