# Supplementary material for: Dendritic-Tumor Fusion Cells Derived Heat Shock Protein70-Peptide Complex Has Enhanced Immunogenicity
Source: PLoS One. 2015 May 11;10(5):e0126075. doi: 10.1371/journal.pone.0126075 (PMC4427282; doi:10.1371/journal.pone.0126075)
Supplement: S4 Statistics — (PDF) [file pone.0126075.s010.pdf]

1. statistics of WB for HSP90/HSP70 ratio Oneway

Descriptives

data

	N	Mean	Std. Deviation	Std. Error	95% Confidence Interval for Mean		Minimum	Maximum
					Lower Bound	Upper Bound		
1.00	3	.6167	.12583	.07265	.3041	.9292	.50	.75
2.00	3	.3033	.02517	.01453	.2408	.3658	.28	.33
Total	6	.4600	.18984	.07750	.2608	.6592	.28	.75

ANOVA

data

	Sum of Squares	df	Mean Square	F	Sig.
Between Groups	.147	1	.147	17.887	.013
Within Groups	.033	4	.008		
Total	.180	5			

2. statistics of WB for HSP110/HSP70 ratio Oneway

Descriptives

group2

	N	Mean	Std. Deviation	Std. Error	95% Confidence Interval for Mean		Minimum	Maximum
					Lower Bound	Upper Bound		
1.00	3	.2500	.05000	.02887	.1258	.3742	.20	.30
2.00	3	.1900	.03606	.02082	.1004	.2796	.15	.22
Total	6	.2200	.05099	.02082	.1665	.2735	.15	.30

ANOVA

group2

	Sum of Squares	df	Mean Square	F	Sig.
Between Groups	.005	1	.005	2.842	.167
Within Groups	.008	4	.002		
Total	.013	5			
